# A multiple regression model of normal central and peripheral motor conduction times

**DOI:** 10.1002/mus.24427

**Published:** 2015-03-14

**Authors:** Stephan R. Jaiser, Jonathan D. Barnes, Stuart N. Baker, Mark R. Baker

**Affiliations:** ^1^ Institute of Neuroscience, Henry Wellcome Building, The Medical School, Newcastle UniversityFramlington Place, Newcastle upon Tyne NE2 4HHUK; ^2^ Department of Clinical NeurophysiologyRoyal Victoria InfirmaryNewcastle upon TyneUK

**Keywords:** central motor conduction time, magnetic stimulation, motor evoked potential, peripheral motor conduction time, regression model

## Abstract

**Introduction:**

The effects of age, height, and gender on magnetic central and peripheral motor conduction times (CMCT, PMCT) were analyzed using a multiple regression model.

**Methods:**

Motor evoked potentials were recorded in 91 healthy volunteers. Magnetic stimulation was performed over the primary motor cortex (cortical latency) and over the cervical and lumbar spines (spinal latency). The spinal latency was taken as an estimate of PMCT and was subtracted from cortical latency to yield CMCT.

**Results:**

Lower limb CMCT correlated significantly with height only; there were no significant predictors for upper limb CMCT. Upper and lower limb PMCT correlated with both age and height.

**Conclusions:**

This is among the largest studies of CMCT in normal subjects. The multiple regression model unifies previously reported simple regression analyses, reconciles past discrepancies, and allows normal ranges to be individualized. *Muscle Nerve* **51**:706–712, 2015

**Abbreviations**
AH: abductor hallucis
APB: abductor pollicis brevis
CMCT: central motor conduction time
CST: corticospinal tract
EDB: extensor digitorum brevis
EDC: extensor digitorum communis
EMG: electromyogram
FDI: first dorsal interosseous
FDS: flexor digitorum superficialis
GC: gastrocnemius
M1: primary motor cortex
MATS: magnetic augmented translumbosacral stimulation
MEP: motor evoked potential
PMCT: peripheral motor conduction time
SEP: somatosensory evoked potential
TA: tibialis anterior

The function of the corticospinal tract (CST) can be assessed non‐invasively using magnetic stimulation. Central motor conduction time (CMCT) has emerged as the most reliable parameter,^1^, ^2^, ^3^ which estimates the conduction time from the primary motor cortex (M1) to spinal motor neurons. Magnetic stimulation is performed over M1 to measure the cortical latency to the target muscle; CMCT is then calculated by subtracting an estimate of the peripheral motor conduction time (PMCT). Clinical CMCT studies commonly estimate PMCT using magnetic stimulation of the spinal roots, as this is well tolerated and avoids the use of a further stimulation modality. We therefore adopted this method for the current study.

Normal ranges of CMCT have been described in several reports (see Table S1 in Supplementary Material, available online). The set of muscles examined was usually small and varied between studies, and results are only in partial agreement where comparisons are possible. This is illustrated by the large spread of mean CMCT to distal muscles: 5.2–8 ms (±0.56–1.7 ms, SD) in the upper limb and 13.4–18.2 ms (±0.9–3.9 ms, SD) in the lower limb. Contributing factors may include small study populations and methodological discrepancies, particularly with regard to the type of stimulator and coil used. In addition, the lower limb representation of M1 was stimulated with a circular coil, whereas a double cone coil is probably better suited to this task, especially for distal muscles.^4^, ^5^

Previous studies have also considered the effect of age, height, and gender on CMCT. Statistical methods ranged from comparisons between discrete groups^6^, ^7^, ^8^, ^9^, ^10^, ^11^ to correlation and regression analysis with individual predictors.^1^^,^ 12, 13, 14, 15 Multiple regression modeling is required to take into account any cross‐correlations between the predictors (e.g., young men are taller on average than older women). This approach has been applied to somatosensory evoked potentials (SEPs),^16^, ^17^, ^18^ but not, to our knowledge, to CMCT.

In this study we sought to clarify the effects of age, height, and gender on CMCT using a stepwise multiple regression model in a large study population stratified by age. PMCT data were modeled similarly. We employed modern equipment and routine clinical methods, including use of standard circular and double cone coils to stimulate the upper and lower limb representations of M1, respectively.

## METHODS

### Subjects

At least 15 volunteers were recruited for each decade of age between 20 and 80 years (50 men and 41 women). Age ranged from 22 to 77 years, and height averaged 171.0 ± 9.6 cm (mean ± SD; range 155.0–188.0 cm). Eighty‐two subjects were right‐handed, and 9 were left‐handed, based on self‐report. None had a history of neurological disorders or diabetes mellitus, contraindications to magnetic stimulation, or used neurotropic medications. All subjects provided written informed consent. The study was approved by the research ethics committee of the Newcastle University Medical Faculty, and conformed to the Declaration of Helsinki.

### Recording

Every effort was made to maintain subjects at a constant level of alertness, and all assessments were carried out on the dominant side. Subjects were seated in a comfortable chair with the arm resting on a cushion. Surface electromyography (EMG) was recorded from abductor pollicis brevis (APB), first dorsal interosseous (FDI), flexor digitorum superficialis (FDS), and extensor digitorum communis (EDC) in the upper limb, and from the extensor digitorum brevis (EDB), abductor hallucis (AH), tibialis anterior (TA), and gastrocnemius (GC) in the lower limb. Adhesive electrodes (Bio‐Logic M0476; Natus Medical, Mundelein, Illinois) were placed in a belly‐tendon montage over the intrinsic muscles of the hand or foot. For the long muscles of the forearm or calf, a belly‐tendon montage would have resulted in large interelectrode distances, thus increasing cross‐talk from neighboring muscles. Hence, electrodes were placed 4 cm apart, one‐third of the distance along the long muscle from its proximal origin. Signals were amplified, band‐pass filtered (30 Hz to 2 kHz; Model D360; Digitimer, Welwyn Garden City, UK) and digitized at 5 kHz (Micro1401; Cambridge Electronic Devices, Cambridge, UK).

### Stimulation

Magnetic stimulation was delivered using a stimulator device (Magstim 200; Magstim Co., Whitland, UK) at a frequency of 0.2 Hz. For upper limb cortical motor evoked potentials (MEPs), a circular coil (13 cm outer diameter) was held over the vertex with its orientation optimized for stimulation of the dominant hemisphere (A side up, left hemisphere; B side up, right hemisphere). For lower limb cortical MEPs, a double cone coil was used in an analogous manner (posterior coil current, left hemisphere; anterior coil current, right hemisphere). Stimulation intensity was set at 10% of maximum stimulator output above the resting motor threshold as defined by the Rossini‐Rothwell method.^19^ Ten MEPs were recorded during a weak background contraction of the target muscles. The background contraction was achieved in the upper limb by opposition of index finger and thumb, and in the lower limb by either dorsiflexion (EDB, TA) or plantarflexion (AH, GC) of ankle and toes. Upper and lower limb root MEPs were recorded at rest with the circular coil centered over the spinous processes of C7 and L1. The range of stimulation intensities used was 35–80% and 40–100% for cortical MEPs of the upper and lower limbs, respectively, and 40–90% and 40–100% for corresponding root MEPs.

### Data Analysis

**Figure 1:**
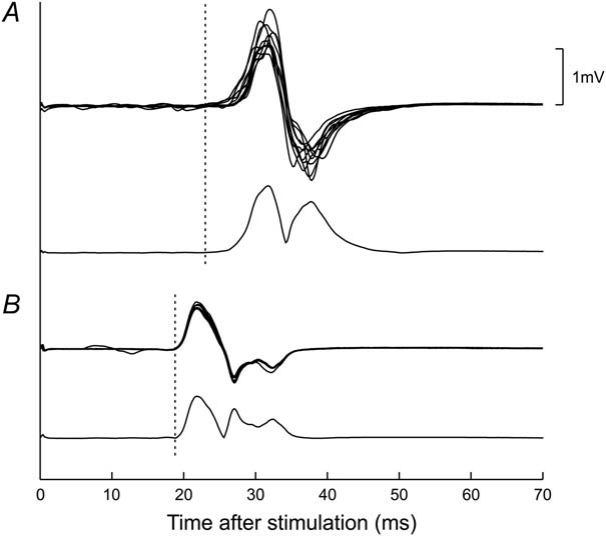
Exemplary single‐subject cortical **(A)** and root **(B)** MEPs in APB. For each site of stimulation, 2 types of trace are shown: 10 superimposed raw sweeps (top) and an average of rectified sweeps (bottom). Latencies (dashed lines) were assigned using the average of the rectified sweeps.

Analysis was performed in MATLAB (The MathWorks, Natick, Massachusetts) using custom scripts. The shortest onset latency for each set of 10 MEPs was assigned interactively. In the presence of a background contraction, the earliest deflection of the MEP with the shortest latency was often ambiguous on superimposed raw traces because of background EMG activity but could be identified easily on averages of rectified MEPs (Fig. 1). Hence, such averages were used to assign latencies throughout.

Stepwise multiple regression models were constructed for all CMCT and PMCT using age, height, and gender as potential predictors (“stepwise” command in MATLAB). Each step involved evaluating the residuals of the model and the associated probability for each predictor and moving a single predictor into or out of the model as recommended by the interactive tool. Significance thresholds were set at ≤0.05 for a predictor to enter the model and at ≥0.10 for it to be removed. The model was considered complete when no further movement of predictors was recommended.

## RESULTS

One subject did not tolerate lower limb cortical MEPs, but all remaining subjects completed all parts of the protocol.

**Figure 2:**
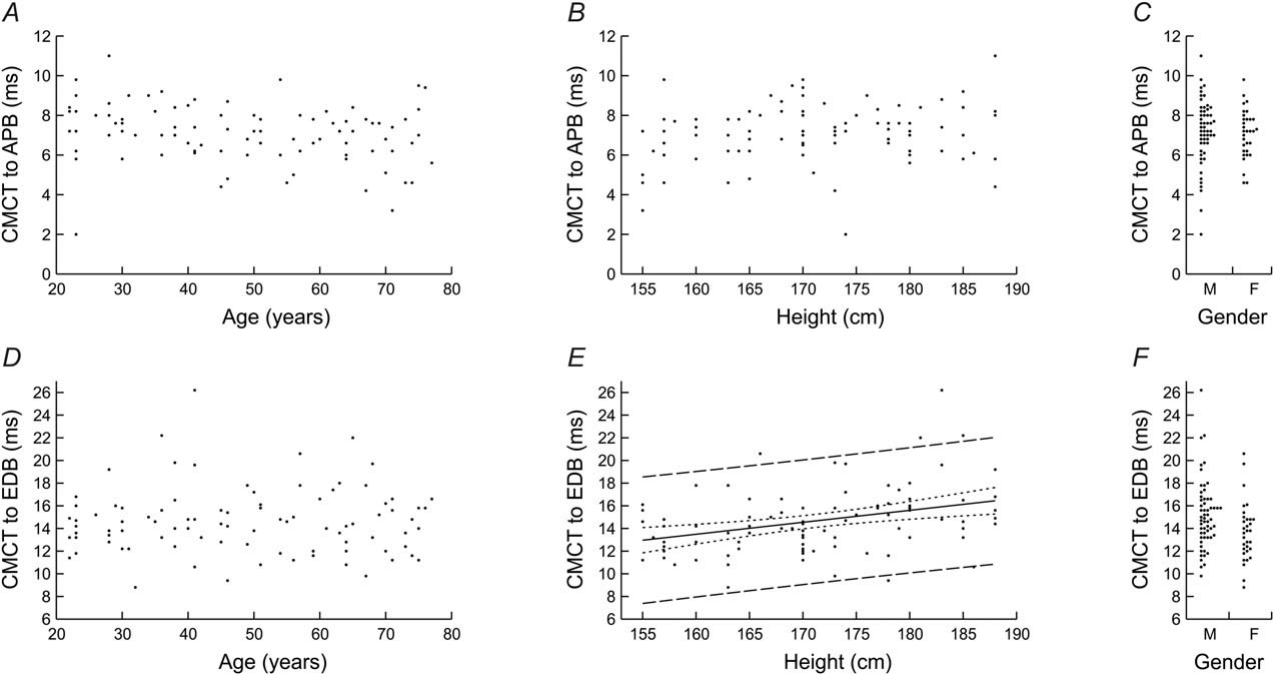
Scatterplots of CMCT to APB **(A–C)** and EDB **(D–F)** against age **(A, D)** and height **(B, E)**, and dot plots of CMCT against gender **(C, F)**. Only the relationship between CMCT to EDB and height was significant. The corresponding regression model is shown (CMCT to EDB = 0.1055 × height − 3.40, *r* ^2^ = 0.123, *P* < 0.001) together with 95% confidence (dotted lines) and prediction (dashed lines) intervals.

**Figure 3:**
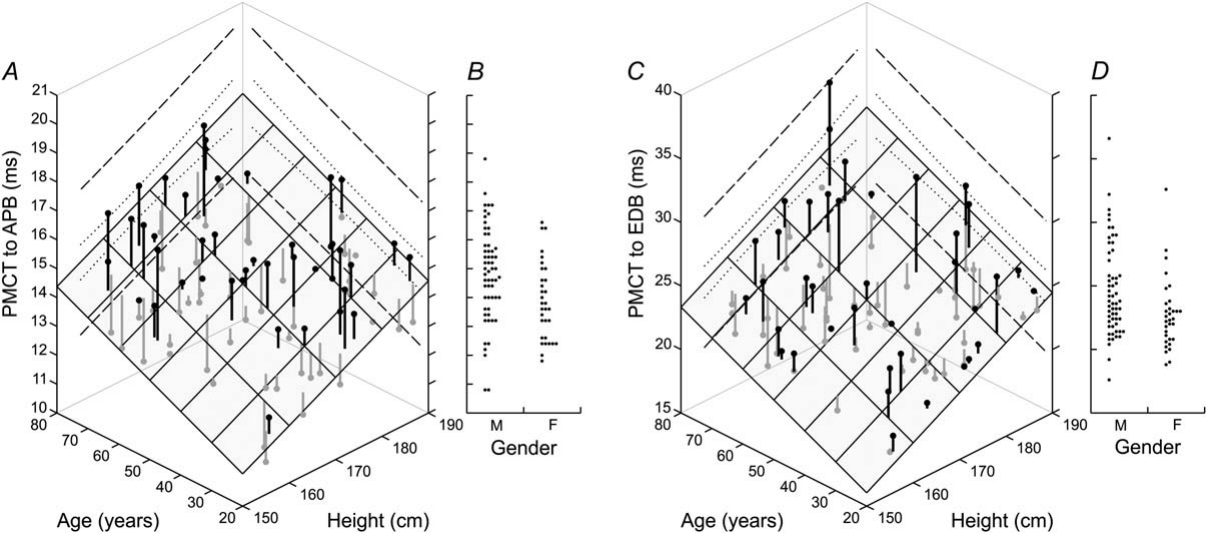
Scatterplots of PMCT to APB and EDB against age and height **(A, C)**, and dot plots of PMCT against gender **(B, D)**. PMCT in both muscles was significantly related to age and height but not gender. The shaded plane shows the regression model (PMCT to APB = 0.0560 × age + 0.0881 × height − 3.28, *r* ^2^ = 0.432, *P* < 0.001; PMCT to EDB = 0.1214 × age + 0.2106 × height − 17.97, *r* ^2^ = 0.482, *P* < 0.001). Vertical lines indicate the residuals of individual data points above (black) and below the plane (gray). Examples of 95% confidence (dotted lines) and prediction intervals (dashed lines) are shown for the upper extremes of age and height.

Means, standard deviations, and regression models are listed numerically in Tables 1 and 2. For a given latency measurement (CMCT or PMCT) and within a given limb, the same predictors were found to be significant across all muscles. Figures 2 and 3 display results for APB and EDB as examples of upper and lower limb muscles.

**Table 1:** Means, SDs, and regression models for CMCT in muscles of upper and lower limbs in this study.

Muscle	Mean ± SD (ms)	Regression model	*r* ^2^	*P*
APB	7.2 ± 1.6	NA	NA	NA
FDI	7.2 ± 1.4	NA	NA	NA
FDS	7.7 ± 2.0	NA	NA	NA
EDC	6.7 ± 1.7	NA	NA	NA
EDB	14.6 ± 2.9	0.1055 × H − 3.40	0.123	<0.001
AH	16.0 ± 3.3	0.0801 × H + 2.29	0.057	0.024
TA	14.5 ± 2.7	0.0919 × H − 1.23	0.107	0.002
GC	15.3 ± 3.7	0.0813 × H + 1.43	0.045	0.044

For upper limb CMCT, no significant regression model could be formulated. H, height (in meters); NA, not applicable.

**Table 2:** Means, SDs, and regression models for PMCT in muscles of upper and lower limbs in this study.

Muscle	Mean ± SD (ms)	Regression model	*r* ^2^	*P*
APB	14.5 ± 1.6	0.0560 × A + 0.0881 × H − 3.28	0.432	<0.001
FDI	15.2 ± 1.6	0.0549 × A + 0.1082 × H − 6.03	0.526	<0.001
FDS	8.7 ± 1.2	0.0373 × A + 0.0412 × H − 0.18	0.291	<0.001
EDC	9.3 ± 1.2	0.0340 × A + 0.0483 × H − 0.62	0.250	<0.001
EDB	23.9 ± 3.5	0.1214 × A + 0.2106 × H − 17.97	0.482	<0.001
AH	25.8 ± 4.0	0.0938 × A + 0.2133 × H − 15.22	0.293	<0.001
TA	13.7 ± 2.4	0.0648 × A + 0.0769 × H − 2.63	0.227	<0.001
GC	15.1 ± 3.3	0.0856 × A + 0.0964 × H − 5.56	0.194	<0.001

A, age (in years); H, height (in meters).

### CMCT

Upper limb CMCT was not significantly related to any of the potential predictors (Table 1 and Fig. 2A–C).

Lower limb CMCT showed a significant positive relationship with height; there was no significant relationship with age or gender. The regression models accounted for approximately 5–12% of the variance observed (*r* ^2^; Table 1). The model for EDB is shown in Figure 2E along with 95% confidence and prediction intervals.

### PMCT

Upper and lower limb PMCT was significantly and positively related to age and height. There was no significant relationship with gender. The regression models explained approximately 19–53% of the variance observed (Table 2), with a tendency for higher r^2^ values for distal compared with proximal muscles. Figure 3A and C illustrates the regression model on a plot of PMCT against age and height. The shaded plane represents the model prediction, with the vertical lines showing the residuals of individual data points above (black) and below the plane (gray). To keep the plot easily interpretable, 95% confidence and prediction intervals are only shown for the upper extremes of age and height.

## DISCUSSION

In this study we have investigated the relationship of CMCT and PMCT with 3 potential predictors chosen for their ready availability. We found that lower limb CMCT depended on height only and that upper limb CMCT was not related significantly to any of the predictors. By contrast, upper and lower limb PMCT both depended on age and height. For each type of latency, the same predictors were consistently significant across all muscles of a given limb, which increases confidence in the individual findings.

### Methods for PMCT Estimation

Several methods are available for estimating PMCT, and the approach used must be kept in mind when comparing the corresponding CMCT readings between studies. Magnetic^20^, ^21^ or electrical^22^ stimulation over the vertebral column excites spinal roots near the exit foramina, and the MEP latency provides an estimate of PMCT. The conduction time along the proximal root segments is not included in PMCT and remains part of CMCT (often called CMCT‐M). This peripheral component of CMCT is particularly pronounced in the lower limbs, where a greater length of the roots is located within the spinal canal. Supramaximal root stimulation, particularly in the lumbosacral territory, can only be achieved with electrical stimulation.^23^ However, this approach causes a greater degree of discomfort than magnetic stimulation and is therefore not in widespread clinical use.

Alternatively, PMCT can be estimated using F‐wave latencies from electrical stimulation of peripheral nerves.^24^ Such PMCT values include the conduction time along the proximal root segments; corresponding CMCT readings (often called CMCT‐F) are shorter and reflect a purer measure of CST conduction than those obtained using root stimulation. Drawbacks of F‐wave latencies include a high inter‐trial variability and the assumption of a fixed turnaround time of 1 ms, which does not take into account that the regenerative volley may be slowed by traveling along a partially refractory axon.^25^ In addition, a different population of motor neurons at different ends of the conduction velocity spectrum may be recruited by the F‐wave and cortical MEPs.^26^

Using a special magnetic augmented translumbosacral stimulation (MATS) coil, Matsumoto and colleagues selectively stimulated either the lumbosacral nerve roots near the exit foramina, akin to conventional magnetic root stimulation, or the conus medullaris within the spinal canal.^27^, ^28^ This makes it possible to estimate the latency from cortex to conus (corticoconus conduction time) and the peripheral component of CMCT‐M (cauda equina conduction time). However, MATS‐based root latencies are relatively low, and corresponding CMCT‐M readings are relatively high when compared with results obtained using conventional coils (see Table S1 in Supplementary Material, available online).^29^, ^30^ Furthermore, root latencies are 0.9 ms shorter than for electrical root stimulation, equivalent to a distance of about 4.5 cm if a nerve conduction velocity of 50 m/s is assumed.^27^ This suggests that the MATS coil excites spinal roots at a more distal point than standard coils, so that CMCT‐M determined using MATS‐based root latencies cannot easily be compared with data obtained using conventional coils.

### CMCT and Height

Our finding of a significant relationship between lower limb CMCT‐M and height is in agreement with several previous studies describing similar results in some^6^ or all muscles under investigation.^1^, ^12^, ^14^ One study reported a trend that did not reach significance.^15^ Furthermore, our regression model for TA (CMCT = 0.0919 × height − 1.23, P = 0.002) concurs with those published previously, particularly with regard to the coefficient for height (CMCT = 0.08 × height − 0.73, P < 0.001^1^; CMCT = 0.083 × height − 0.47, P < 0.0001^14^).

It has been suggested that this relationship may be attributable to the peripheral component of CMCT‐M. If so, no such relationship should exist for CMCT‐F. Although several studies have reported both CMCT‐M and CMCT‐F, few have considered how both types of CMCT may differ in their relationship to height. One report showed that CMCT‐M was related to height in 1 of 3 muscles, but CMCT‐F was not^6^; another study identified a significant correlation of CMCT‐M with height, without commenting on CMCT‐F data.^14^ A further study did not make it clear whether both types of CMCT were analyzed in a separate or pooled fashion.^15^ Recently, a study described height as uncorrelated with lower limb corticoconus conduction time but correlated significantly with CMCT‐M and cauda equina conduction time.^29^ However, the use of a MATS coil for root stimulation would have exaggerated the peripheral component of CMCT‐M. It therefore remains unclear to what extent the peripheral component underlies the correlation of height and conventional lower limb CMCT‐M. This could be addressed by a study in which the conus is stimulated with a MATS coil and the roots with a standard coil, thus allowing measurement of corticoconus conduction time as well as true conventional CMCT‐M and its peripheral component.

There is consensus that upper limb CMCT does not correlate with height.^1^^,^ 12, 13, 14, 15 This may be because of the shorter proximal root segments in the cervical spine, as height relates less strongly with the length of the CST to the upper limb,^1^, ^12^ or both.

### CMCT and Age

The effect of age on CMCT is controversial. We observed no correlation between age and CMCT‐M. This concurs with 6 earlier reports measuring both CMCT‐M and CMCT‐F and finding that neither of them were significantly related to age.^1^, ^6^, ^9^, ^11^^,^ 13, 15 However, 3 other studies reported a significant positive relationship. The first employed a MATS coil and, although corticoconus conduction time did not correlate with age, CMCT‐M and cauda equina conduction time did show a correlation. The reported correlation of CMCT‐M with age is explicable in terms of an exaggerated peripheral component.^30^ A second investigation calculated PMCT from F‐wave latencies in such a way that a peripheral component of unclear magnitude remained part of the CMCT‐F.^7^, ^30^ The final study compared CMCT‐M in 2 muscles between 3 groups of different ages that had been matched for height but did not make any adjustment for multiple comparisons.^8^ Thus, whenever a significant relationship between CMCT and age was reported, it could usually be attributed to an increased peripheral component. By contrast, the peripheral component of conventional CMCT‐M appears to be sufficiently small to avoid giving rise to a significant relationship.

### CMCT and Gender

Similar to our findings, previous investigations showed CMCT to be unaffected by gender,^8^, ^13^ or any differences between men and women were attributed to height differences between the genders.^6^, ^10^, ^12^, ^14^

### PMCT

It is well known that age and height correlate negatively with peripheral nerve conduction velocities and correlate positively with distal motor and F‐wave latencies, whereas gender is generally not considered a significant predictor.^31^, ^32^ Similarly, PMCT is related to age^6^, ^8^, ^9^, ^30^ and height.^6^, ^12^, ^14^ The proportion of variance explained by our model (r^2^) was greater for PMCT than for CMCT. We are not aware of any previous multiple regression models for PMCT, but the proportion of variance explained by our model for PMCT is in broad agreement with values reported for multiple regression models of related peripheral conduction parameters.^31^, ^32^

The relationship between PMCT and height is explained readily by the strong correlation between height and limb length and thus the length of the peripheral nerves.^33^ The observation that PMCT, but not CMCT, correlates with age may be attributable to the greater exposure of peripheral nerves to minor trauma and injuries.^30^ Indeed, aging is not only known to cause subclinical peripheral nerve lesions at common entrapment sites,^34^ but also leads to progressive loss of motor units, particularly affecting the largest and fastest units.^35^

CMCT and PMCT showed greater spread in the lower limb than in the upper limb (Tables 1 and 2). This may be attributable to variability of the point of stimulation along the cauda equina and exiting nerve roots. This hypothesis is consistent with the larger variability of CMCT and PMCT observed in distal compared with proximal lower limb muscles, because distal muscles have longer root segments within the cauda equina.

### Clinical Application

In clinical practice, numerical results are typically compared with normal ranges or cut‐off values, which constitutes fixed‐level testing at a predetermined significance level. Here, an appropriate cut‐off would be the upper bound of a chosen prediction interval. The bound can be approximated by evaluating the regression model with the parameters of the patient and adding *q*_1 − α/2_ standard deviations, where α is the desired significance level and *q* is the inverse of the normal cumulative distribution function. For example, a 95% prediction interval has α = 0.05 and *q*_1 − α/2_ = *q*_0.975_ = 1.960; this upper bound would be exceeded by α/2 = 0.025 = 2.5% of normal readings.

Alternatively, we can evaluate the probability of observing a latency at least as high as that of the patient under the null hypothesis that the latency of the patient is normal. The regression model is evaluated with the parameters of the patient, and the *Z*‐score is calculated as *Z* = (actual result – regression result) / standard deviation of residuals. The corresponding probability is then computed as ϕ(−|*Z*|), where ϕ is the cumulative normal distribution.

The data provided allow either approach to be implemented easily; we have deliberately not provided prescribed cut‐off values, as they would force the reader into fixed‐level testing with a chosen significance level.

In conclusion, this is among the largest studies of CMCT‐M in normal subjects and, to our knowledge, the only study to employ multiple regression modeling. Such an approach was applied to SEPs more than 3 decades ago, and its application to MEP data has helped to reconcile controversies surrounding the effects of age and height on CMCT. In addition, the model accounts for 5–12% (CMCT) or 19–53% (PMCT) of variance. Paired with side‐to‐side comparisons within a given subject, this should boost the diagnostic accuracy and precision of CMCT‐M.

## Supporting information

Supplementary Information
