# Supplementary material for: A multiple regression model of normal central and peripheral motor conduction times
Source: Muscle Nerve. 2015 Mar 14;51(5):706–12. doi: 10.1002/mus.24427 (PMC4858813; doi:10.1002/mus.24427)
Supplement: Supplementary file 1 — Supplementary Information [file MUS-51-706-s001.docx]

**Supplementary Table 1.** Previously reported normal CMCT and PMCT data (mean ± standard deviation, SD). Studies have only been included if a facilitatory background contraction was maintained during cortical stimulation and if PMCT was estimated using magnetic root stimulation. All used circular coils in conjunction with the stimulators listed, but other methods, including coil size, were described in variable detail and cannot be compared easily. Results are limited to the set of muscles considered in our study; we did not find any reported data for FDS. Two studies categorized some results by age or height without giving pooled data, so results are listed separately for each group ^6,8^. One report provided an upper limit of normal as mean + 3SD without individually stating the mean or SD ^3^, and another specified only CMCT but not PMCT ^7^ (NS=not specified).

| **Muscle** | **CMCT (ms)** | **PMCT (ms)** | **Stimulator** | **N** | **Reference** |
| --- | --- | --- | --- | --- | --- |
| APB | 6.88 ± 0.56 | 13.12 ± 1.35 | Magstim 200 | 30 | ^35^ |
|  | 8.0 ± 1.2 | 13.1 ± 1.0 | Own design | 27 | ^36^ |
|  | 5.2 ± 0.6 | 15.6 ± 1.2 | Dantec or Magstim 200 | 53 | ^13^ |
|  | 6.7 ± 1.2 | NS | Dantec | 95 | ^7^ |
|  | 6.73 ± 1.01 | 13.58 ± 0.98 | Magstim 200 | 30 | ^15^ |
|  | 6.7 ± 1.7 (age 31.2 ± 16.8)  6.3 ± 1.0 (age 78.7 ± 4.8) | 11.1 ± 0.7 (age 31.2 ± 16.8)  11.7 ± 0.9 (age 78.7 ± 4.8) | Own design | 14  26 | ^9^ |
|  | 8.0 ± 1.2 | 11.8 ± 1.0 | Own design | 30 | ^37^ |
| FDI | 6.0 ± 1.0 | 14.6 ± 1.3 | Magstim 200 | 57 | ^38^ |
|  | 5.8 ± 1.0 (age ≤29)  6.0 ± 0.9 (age 30-59)  6.5 ± 1.1 (age ≥60) | 14.0 ± 1.3  14.6 ± 1.3  14.9 ± 1.4 | Magstim 200 | 57 | ^8^ |
| EDC | 5.6 ± 0.9 | 9.1 ± 0.8 | Magstim 200 | 57 | ^38^ |
|  | 6.4 ± 1.2 | NS | Dantec | 42 | ^7^ |

| **Muscle** | **CMCT (ms)** | **PMCT (ms)** | **Stimulator** | **n** | **Reference** |
| --- | --- | --- | --- | --- | --- |
| EDB | 13.4 ± 1.7 (pooled) | 23.9 ± 2.0 (height 150-174cm)  25.4 ± 1.9 (height 175-191cm) | Dantec or Magstim 200 | 46 | ^6^ |
|  | 14.53 ± 1.50 | 21.71 ± 1.92 | Magstim 200 | 30 | ^15^ |
|  | 15.7 ± 2.4 (age ≤29)  15.9 ± 2.0 (age 30-59)  18.2 ± 3.9 (age ≥60) | 24.8 ± 1.8  23.3 ± 2.6  23.9 ± 2.8 | Magstim 200 | 57 | ^8^ |
| AH | 16.7 ± 2.4 | 24.5 ± 2.1 | Own design | 27 | ^37^ |
|  | 15.9 ± 2.0 | 24.3 ± 2.6 | Magstim 200 | 57 | ^39^ |
|  | 18.2 (mean + 3SD) | 30.1 (mean + 3SD) | Magstim 200 | 30 | ^3^ |
|  | 16.9 ± 0.9 | 23.3 ± 2.5 | Magstim 200 | 15 | ^40^ |
| TA | 14.35 ± 0.85 | 11.73 ± 1.37 | Magstim 200 | 30 | ^36^ |
|  | 14.3 ± 1.7 | 14.7 ± 1.8 | Magstim 200 | 57 | ^39^ |
|  | 14.8 ± 1.1 | 11.7 ± 1.1 | Magstim 200 | 52 | ^12^ |
|  | 17.1 (mean + 3SD) | 16.1 (mean + 3SD) | Magstim 200 | 30 | ^3^ |
|  | 12.8 ± 1.4 (height 150-174cm)  14.0 ± 1.3 (height 175-191cm) | 16.1 ± 2.3 (pooled) | Dantec or Magstim 200 | 46 | ^6^ |
|  | 13.8 ± 1.5 | 12.3 ± 1.2 | Magstim 200 | 50 | ^14^ |
|  | 14.23 ± 1.71 | 13.22 ± 1.19 | Magstim 200 | 30 | ^15^ |
|  | 13.4 ± 1.9 (age ≤29)  14.3 ± 1.7 (age 30-59)  16.1 ± 1.9 (age ≥60) | 14.7 ± 1.3  14.7 ± 2.1  15.5 ± 2.0 | Magstim 200 | 57 | ^8^ |
|  | 14.6 ± 1.2 | 11.5 ± 0.9 | Magstim 200 | 51 | ^29^ |
|  | 14.7 ± 1.3 | 11.5 ± 1.1 | Magstim 200 | 100 | ^30^ |
|  | 15.3 ± 1.0 | 12.7 ± 1.6 | Own design | 30 | ^38^ |
| GC | 14.2 ± 1.5 | 13.4 ± 1.0 | Magstim 200 | 57 | ^39^ |
